# Isolated Left Pulmonary Artery Agenesis: A Case Report

**DOI:** 10.4274/Mirt.7

**Published:** 2012-08-01

**Authors:** Tansel Ansal Balcı, Zehra Pınar Koç, Gamze Kırkıl, Ahmet Kürşad Poyraz

**Affiliations:** 1 Fırat University Faculty of Medicine Department of Nuclear Medicine, Elazığ, Turkey; 2 Fırat University Faculty of Medicine, Department of Pulmonary Disease, Elazığ, Turkey; 3 Fırat University Faculty of Medicine, Department of Radiology, Elazığ, Turkey

**Keywords:** Pulmonary artery, agenesis, ventilation-perfusion scintigraphy, X-Ray computed tomography

## Abstract

Unilateral pulmonary artery agenesis without any cardiovascular malformation is a rare anomaly. We present the imaging findings of a patient who was diagnosed as isolated left pulmonary artery agenesis. A 27-year-old female patient was admitted to our hospital due to dyspnea during exercise for five years. Chest X-ray revealed minimally small left pulmonary hilum and left lung. She was admitted to our clinic with the suspicion of pulmonary artery pathology. Absent perfusion of the left lung with normal ventilation was visualized on scintigraphy. MDCT angiography of pulmonary arteries showed absent left main pulmonary artery with systemic collaterals around left hemithorax. Pulmonary artery agenesis can be asymptomatic and isolated until adulthood. Both scintigraphy and CT angiography images of pulmonary artery agenesis of a patient are rare in the literature. Pulmonary ventilation-perfusion scintigraphy can be used not only for pulmonary embolism but also pathologies involving pulmonary artery and its branches.

**Conflict of interest:**None declared.

## INTRODUCTION

Isolated unilateral absence of pulmonary artery is a rare congenital malformation. It is a rare anomaly especially for adults, asymptomatic in 30% of patients or presented with mild symptoms at adulthood (1,2,3,4). Although generally left sided agenesis is presented with congenital cardiac malformations and right sided agenesis is usually isolated, our patient had isolated left sided pulmonary artery agenesis without other anomaly (2,5,6). We present the chest radiography, ventilation-perfusion scintigraphy and computed tomography (CT) angiography images of this rare malformation. 

## CASE REPORT

**Figure 1:**
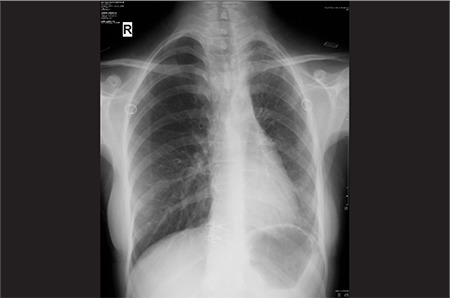
Chest radiograph shows small sized left lung and slight increase of interstitial markings on left. Right to left mediastinal shift and hypertrophic right lung is also seen

A 27-year-old female patient was admitted to our hospital due to dyspnea during exercise. She had been suffering from exertional dyspnea for 5 years. The patient did not have a family history of congenital anomalies. Physical examination findings, biochemistry parameters and vital signs were in normal range. There was no sign of a respiratory disease in physical examination. Patient’s respiratory function tests and echocardiographic evaluation were also normal. Chest radiograph (Figure 1) revealed minimally decreased left lung volume with increased basal reticular opacities. Right to left mediastinal shift and hyperlucent and hypertrophic right lung was accompanying. Because of the X-ray findings, the patient was referred for ventilation-perfusion scintigraphy with the suspicion of arterial pathology. Perfusion scintigraphy was performed with the intravenous administration of 5 mCi Tc-99m macroaggregated albumin (MAA) and anteroposterior, left and right lateral and oblique projection planar images of the chest region with a double head SPECT gamma camera (GE Infinia, Israel) equipped with a low energy high resolution collimators was performed. After inhaler administration of 25 mCi Tc-99m dietilen triamine pentaacetic acide (DTPA) with the same equipment at the same projections. Absent perfusion of the left lung on the perfusion scintigraphy and normal ventilation with minimally decreased volume of left lung on the ventilation scintigraphy were visualized (Figure 2a, 2b). Although V/Q scanning provided strong evidence of the pulmonary artery agenesis the diagnosis was also confirmed by CT angiography. 

Multidetector row computed tomography (MDCT) angiography of pulmonary arteries (Figure 3a, 3b) revealed absence of left main pulmonary artery. Blood supply was achieved via left subdiaphragmatic, intercostal and bronchial arterial collateral branches. Diameter of pulmonary truncus and right main pulmonary artery were in normal range. Left lung volume was decreased and interlobular septal thickening was prominent in basal lung. Right lung volume was increased. 

## LITERATURE REVIEW AND DISCUSSION

Because of limited reports and asymptomatic patients, the prevalence of unilateral pulmonary artery agenesis is unclear. Minority (15-30%) of patients with unilateral pulmonary artery agenesis are asymptomatic. Chest radiograph may give clue of pulmonary artery agenesis. However pulmonary hypoplasia has the similar chest radiograph as well. Unilateral pulmonary artery agenesis and hypoplasia should be especially suspected in case of small hemithorax and ipsilateral small pulmonary hilum on chest radiograph (7). Confirmation and anatomical details (i.e. presence of collateral arteries, pulmonary hypertension, great vessel abnormalities, cardiac malformations and parenchymal lung diseases) can be discerned by computed tomography, magnetic resonance imaging and scintigraphy (2,8,9,10,11). Unilateral absence of pulmonary artery is usually diagnosed and surgically treated in the first year of life if it is accompanied to cardiovascular malformations (1). However, isolated pulmonary artery agenesis can be asymptomatic and a diagnosis may not be made until they reach adulthood (8,12). 

With the most recent review published in 2002, totally 108 cases of isolated pulmonary artery agenesis not accompanied by cardiac anomalies have been reported (8). According to this review only 14 of these cases were asymptomatic and the median age of detection was 14. Thirty seven percent of these cases had history of frequent pulmonary infections. Pulmonary edema was observed in 12% and in some of patients, respiratory insufficiency developed in the later stages. Exercise limitations were described as 40%. Our patient has only exertional dyspnea for only 5 years without another complaint. In addition, the most frequently performed diagnostic procedures were as the follows: chest radiography, ventilation-perfusion scanning, cardiac catheterization (including pulmonary venous wedge angiography), echocardiography, and CT scanning or MRI.

Chest X-ray findings are mild for this anomaly and it is difficult to diagnose these patients with just X-ray. The most common finding is the hyperlucency of healthy side (13). 

There are several cases of single pulmonary artery aplasia and associated anomalies (13,14,15,16,17). Perfusion scintigraphy has been performed to some of these cases and authors mentioned about the importance of this technique for diagnosis of this anomaly (14,16). Ventilation-perfusion studies in pulmonary agenesis are typically described as showing no perfusion on the affected side with intact or diminished ventilation (9,11,18). Although they are slightly decreased, perfusion and ventilation are intact in pulmonary hypoplasia as the differential diagnosis (7). The perfusion scintigraphy is directly associated with the perfusion of lung and that’s why the clear visualization of perfusion anomalies is possible by means of this technique. Previous chest CT also was unluckily reported as normal by another medical center where the patient was admitted with the same complaint. Pulmonary agenesis is one of the rare false positive results for pulmonary embolism as in our patient (19,20). CT angiography was performed to the patient with the information obtained from perfusion scintigraphy and the absence of left pulmonary artery was confirmed. 

In conclusion, in the diagnosis of pulmonary artery agenesis chest x-ray is a first step diagnostic tool. In case of a hypoplastic hemithorax and small ipsilateral hilum with diminished pulmonary vascularity, pulmonary artery agenesis should be suspected. Computed tomography is generally sufficient for definitive diagnosis and provides detailed morphological information and determines the presence of cardiovascular malformations. Scintigraphy can be preferred to see the exact perfusion status and for presurgical planning. Also, echocardiography should be performed to exclude intracardiac anomalies in these patients. 

## Figures and Tables

**Figure 2a:**
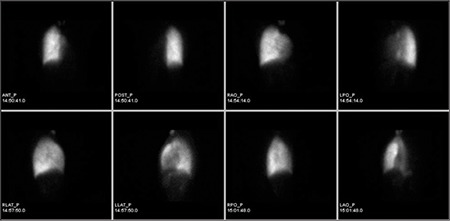
Anteroposterior, left and right lateral, left and right posterior oblique, left and right anterior oblique images of Tc-99m macroaggregated albumin (MAA) perfusion scintigraphy shows absent left lung perfusion

**Figure 2b:**
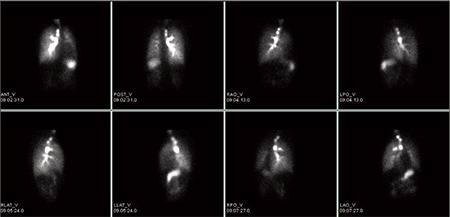
Anteroposterior, left and right lateral, posterior and anterior oblique images of Tc-99m DTPA aerosol ventilation scintigraphy shows minimally decreased ventilation on left lung

**Figure 3a:**
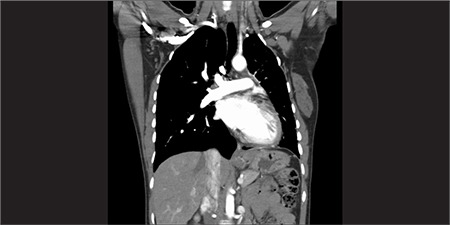
Coronary oriented maximum-intensity-projection image from CT angiography shows small left hemithorax and absence of left main pulmonary artery. Note the left subdiaphragmatic collateral branches

**Figure 3b:**
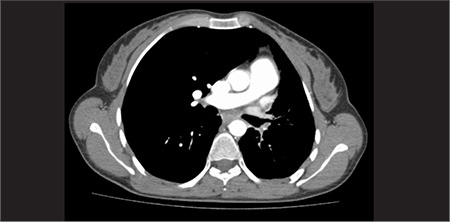
Axial oriented maximum-intensity-projection image from CT angiography shows small left hemithorax, mediastinal shift and absence of the left main pulmonary artery with left hilar bronchial artery collaterals
